# RECK-Mediated β1-Integrin Regulation by TGF-β1 Is Critical for Wound Contraction in Mice

**DOI:** 10.1371/journal.pone.0135005

**Published:** 2015-08-06

**Authors:** Jaime Gutiérrez, Cristian A. Droppelmann, Osvaldo Contreras, Chiaki Takahashi, Enrique Brandan

**Affiliations:** 1 Cellular Signaling and Differentiation Laboratory (CSDL), School of Medical Technology, Health Sciences Faculty, Universidad San Sebastian, Santiago, Chile; 2 Centro de Regeneración y Envejecimiento (CARE), Departamento de Biología Celular y Molecular, Facultad de Ciencias Biológicas, Pontificia Universidad Católica de Chile, Santiago, Chile; 3 Molecular Medicine Group, Robarts Research Institute, Western University, London, Ontario, Canada; 4 Oncology and Molecular Biology, Cancer and Stem Cell Research Program, Cancer Research Institute, Kanazawa University, Kanazawa, Japan; Fox Chase Cancer Center, UNITED STATES

## Abstract

Fibroblasts are critical for wound contraction; a pivotal step in wound healing. They produce and modify the extracellular matrix (ECM) required for the proper tissue remodeling. Reversion-inducing cysteine-rich protein with Kazal motifs (RECK) is a key regulator of ECM homeostasis and turnover. However, its role in wound contraction is presently unknown. Here we describe that Transforming growth factor type β1 (TGF-β1), one of the main pro-fibrotic wound-healing promoting factors, decreases RECK expression in fibroblasts through the Smad and JNK dependent pathways. This TGF-β1 dependent downregulation of RECK occurs with the concomitant increase of β1-integrin, which is required for fibroblasts adhesion and wound contraction through the activation of focal adhesion kinase (FAK). Loss and gain RECK expression experiments performed in different types of fibroblasts indicate that RECK downregulation mediates TGF-β1 dependent β1-integrin expression. Also, reduced levels of RECK potentiate TGF-β1 effects over fibroblasts FAK-dependent contraction, without affecting its cognate signaling. The above results were confirmed on fibroblasts derived from the *Reck* ^+/-^ mice compared to wild type-derived fibroblasts. We observed that *Reck* ^+/-^ mice heal dermal wounds more efficiently than wild type mice. Our results reveal a critical role for RECK in skin wound contraction as a key mediator in the axis: TGF-β1—RECK- β1-integrin.

## Introduction

Wound healing is a complex and dynamic process, which involves the coordinated action of different cell types, promoting hemostasis, wound contraction and remodeling [1, 2]. Fibroblasts are one of the key players in wound healing. They are involved in breaking down the fibrin clot; produce and remodel the extracellular matrix (ECM), which is required to support angiogenesis; granulation-tissue generation; and re-epithelialization [1, 3]. In addition, fibroblasts are essential for wound contraction [3, 4]. All of these fibroblast activities are controlled by different cytokines and chemokines, such as transforming growth factor β (TGF-β1), angiotensin II (Ang II), connective tissue growth factor (CTGF/CCN-2), and endothelin-1 (ET-1), all of which are mainly produced by mesenchymal and inflammatory cells [5–7]. TGF-β1 is of particular importance as it has previously been described as one of the main pro-fibrotic wound-healing promoting factors [8, 9].

TGF-β1 induces fibroblasts to express ECM components, such as collagens and fibronectin (FN) [8, 10], and simultaneously regulates the expression of matrix metalloproteinases (MMPs) [10, 11], which are zinc and calcium dependent endopeptidases associated with different processes that require modification of the ECM [2, 12]. TGF-β1 also stimulates fibroblasts to express integrin subunit β1 (β1-integrin) [13, 14], which is essential for fibroblasts adhesion, migration, ECM contraction [15] and wound healing [16]. The integrin dependent adhesion to fibrous proteins in the ECM triggers the formation actin stress fibers [17, 18], which anchor the cytoskeleton to the ECM through a cell membrane protein complex at focal adhesion sites (FA) [19, 20]. An important component of the FA is focal adhesion kinase (FAK), a key component of the signal transduction pathways activated by integrins in focal contacts [19, 21]. Altogether, these changes induced by TGF-β1 produce a specialized transdifferentiation of fibroblasts to myofibroblasts [13, 22], which is characterized by the expression of smooth muscle α-actin (α-SMA) giving them contractile properties [23, 24].

Reversion-inducing cysteine-rich protein with Kazal motifs (RECK) is a membrane GPI-anchored glycoprotein that acts as an inhibitor of several metalloproteinases which makes it a key regulator of ECM remodeling and integrity [25, 26]. Homozygous *Reck* knockout mice (*Reck* ^-/-^) die *in utero*, showing massive hemorrhage and smaller body size compared with wild type mice, while heterozygous *Reck* knockout mice (*Reck* ^+/-^) are fertile and show no obvious phenotype [26]. Histological examination of *Reck* ^/-^ embryos has shown unstructured mesenchymal tissues with nearly absent collagen arrays and abnormal organogenesis within the embryos, indicating the key role of RECK in ECM integrity and homeostasis [26, 27]. Previous studies have also shown that RECK depletion is related to decreased β1-integrin activation and diminished FAK activity in human endothelial cells, which is associated with decreased proliferation and a defective capacity to form vascular tubes [28]. This indicates that RECK regulates the β1-integrin activity dependent signaling [28, 29]. Although the aforementioned studies strongly suggest that RECK plays a role in fibroblast contraction and wound closure, this potential function of RECK has currently not been evaluated.

In this study, we demonstrate that TGF-β1, through its canonical Smad and non-canonical JNK dependent pathways [30], decreases RECK levels in fibroblasts, concomitant with an increase of β1-integrin levels. Moreover, the TGF-β1 dependent induction of β1-integrin requires the downregulation of RECK. Fibroblasts obtained from *Reck* ^+/-^ mice express more β1-integrin and show increased contraction properties compared to wild type fibroblasts. We also demonstrate that *Reck* ^+/-^ mice heal dermal wounds more efficiently than wild type mice. Our results show a critical role for RECK in wound contraction and reveal a new pathway regulating wound contraction, the axis: TGF-β1—RECK- β1-integrin.

## Results

### TGF-β1 reduces RECK expression levels in fibroblasts

**Fig 1:**
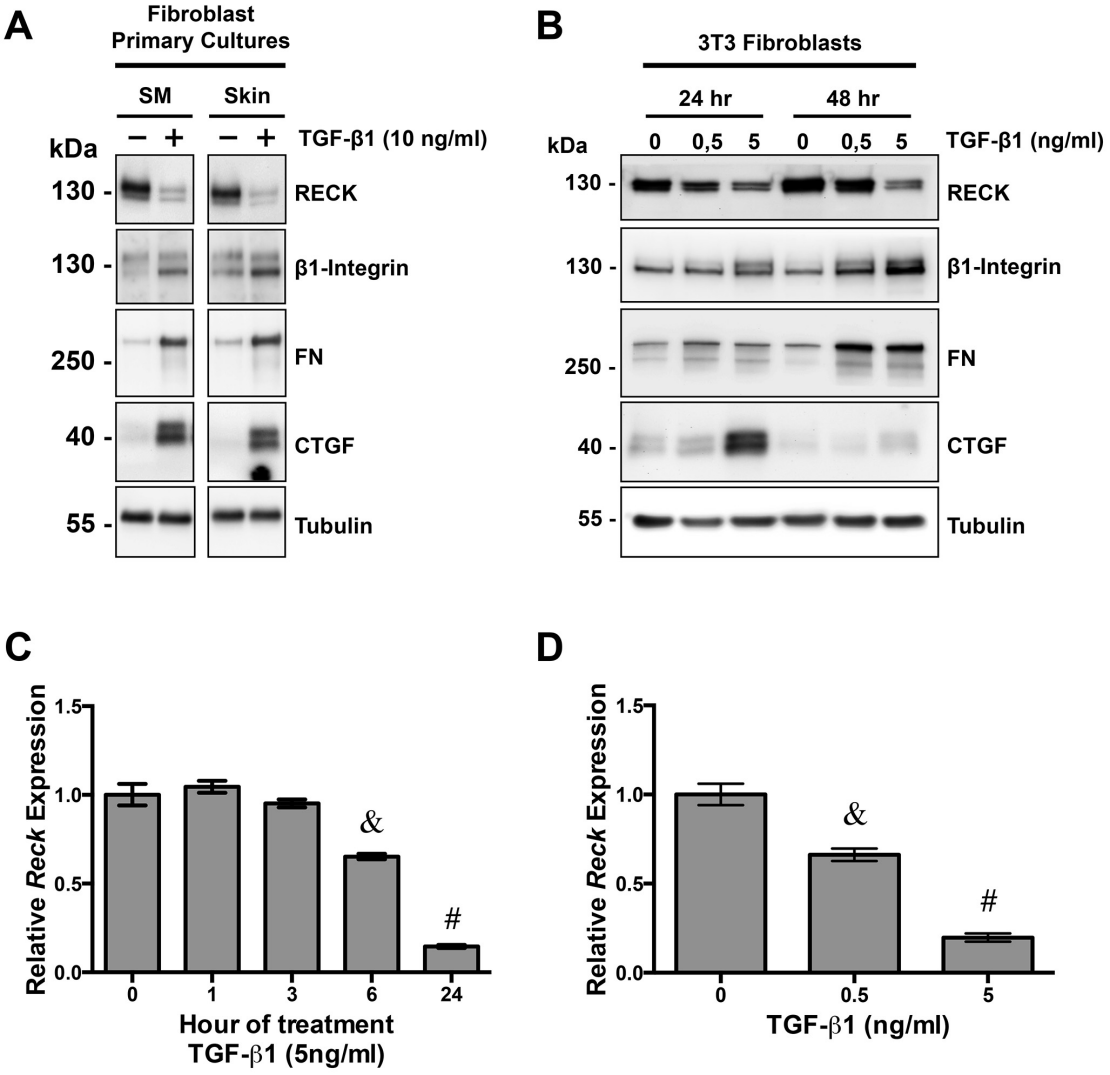
TGF-β1 decreases RECK expression in fibroblasts. **(A)** NIH3T3 fibroblasts and were incubated with TGF-β1 for 24 and 48 h at the indicated concentrations. Western blots analysis of cell extracts were performed to determine the expression of RECK, β1-integrin, FN and CTGF. β-tubulin (Tubulin) levels were used as a loading control. **(B)** Primary fibroblasts cultures derived from tibialis anterior skeletal muscles (SM) and skin biopsies of 3-month-old WT mice were incubated with TGF-β1 for 24 as indicated. Western blot analysis of cell extracts were performed to determine the levels of RECK, FN, and CTGF. tubulin levels were used as a loading control. **(C)** NIH3T3 fibroblasts were incubated with 5 ng/ml of TGF-β1 for the indicated times. At the end of the assay, total RNA was extracted and was reverse transcribed into complementary DNA. Taqman quantitative real-time PCR was performed to determine *Reck* expression. mRNA expression was quantified using the comparative ΔC_T_ method (2^-ΔΔCT^) using *Gadph* as a reference gene. mRNA levels are presented relative to the mean expression of the control (untreated cells). **(D)** *Reck* mRNA expression in NIH3T3 fibroblasts incubated with different concentrations of TGF-β1 for 24 hours was determined as in (C). In C and D, values are expressed as mean +/- standard deviation (SD) of two independent experiments. In C, &, P<0.05 relative to 0 hour; #, P<0.05 relative to 6 hours. In D, &, P<0.05 relative to 0 ng/ml; #, P<0.05 relative to 0,5 ng/ml.

To evaluate if TGF-β1 regulates the expression of RECK in fibroblasts, mouse primary fibroblast cultures, derived from *tibialis anterior* skeletal muscle (SM) or skin biopsies, were treated with TGF-β1 at the indicated concentration. Fig 1A, shows that TGF-β1 reduces the amount of RECK after 24 hours. This effect was concomitant to the induction of three known proteins positively regulated by TGF-β1: the β1 integrin sub-unit, the ECM protein FN, and the pro-fibrotic growth factor CTGF [31–33]. These effects were reproduced in the fibroblast cell line NIH3T3, as shown in Fig 1B. Here, TGF-β1 reduces the amount of RECK after 24 or 48 hours of incubation in a concentration dependent manner, concomitant to the induction of β1 integrin sub-unit, FN and CTGF [31–33]. The latter is transiently upregulated by TGF-β1, peaking at 24 h and diminishing thereafter, as previously shown [31].

To evaluate if the TGF-β1 dependent downregulation of RECK in cultured fibroblasts is at the transcriptional level, we analyzed the levels of *Reck* mRNA by qPCR in NIH3T3 fibroblasts treated with TGF-β1. Fig 1C, shows that the levels of *Reck* mRNA were progressively downregulated from 6 to 24 hours in response to TGF-β1. Moreover, the effect was TGF-β1 concentration-dependent, as shown in Fig 1D.

### TGF-β1 reduces RECK expression levels in fibroblasts through a JNK and Smad dependent pathways

**Fig 2:**
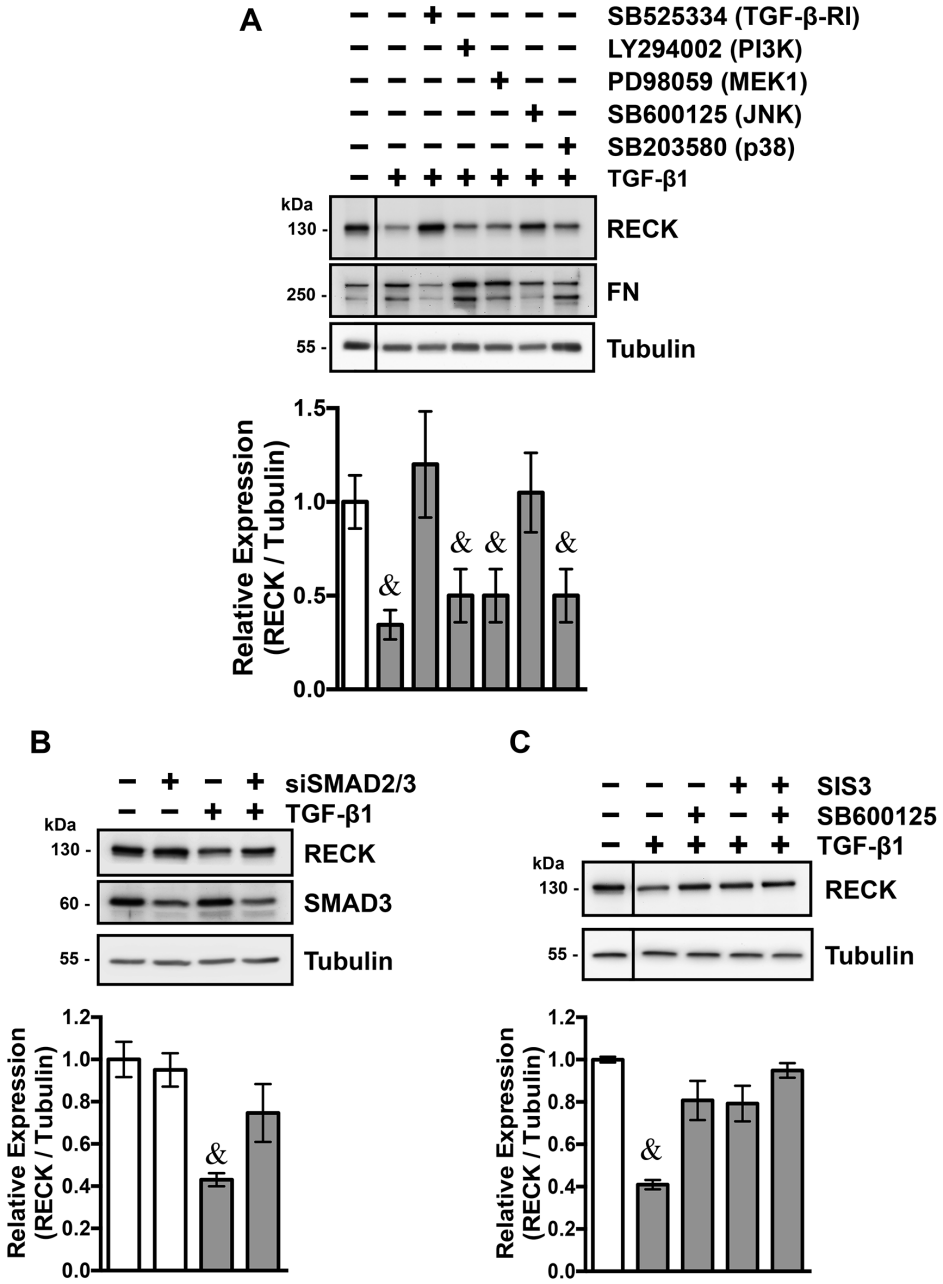
TGF-β1 decreases RECK levels through a Smad and JNK dependent *pathway*. **(A)** NIH3T3 fibroblasts were pre-treated for 30 minutes with different inhibitors: TGF-β-RI kinase inhibitor SB525334, PI3K inhibitor LY294002, MEK1 inhibitor PD98059, JNK inhibitor SB600125, and p38 inhibitor SB203580. After the pre-treatment, fibroblasts were treated with 5ng/ml of TGF-β1 for 24 hours or untreated to serve as controls. Western blot analysis of cell extracts were performed to determine the levels of RECK and FN. tubulin levels were used as a loading control. **(B)** NIH3T3 fibroblasts were transiently transfected with a pool of siRNA against Smad-2 and Smad-3 or with a scrambled siRNA as a control. 24 h post-transfection, cells were treated with 5 ng/ml of TGF-β1 for 24 h, or left untreated to serve as a control. Western blot analysis of cell extracts were performed to determine the levels of RECK, Smad-3 and β1-Integrin. β-tubulin levels were used as a loading control. **(C)** NIH3T3 fibroblasts were pre-treated for 30 minutes with SIS3, a specific inhibitor of Smad-3 activation, and the JNK inhibitor SB600125; cells were treated either alone or in combination. Afterwards, fibroblasts were treated with 5 ng/ml of TGF-β1, or left untreated as a control, for 24h. Western blot analysis of cell extracts were performed to determine the levels of RECK and β1-Integrin. β-tubulin levels were used as a loading control. The quantifications shown in A, B and C are from two independent. Statistical significance was assessed using two-way ANOVA and a Bonferroni multiple-comparison post hoc test. &, P<0.05 relative to TGF-β1 untreated fibroblasts.

TGF-β1 activates the canonical Smad-2 and -3 dependent pathways and the non-canonical PI3K, MEK-1 MAPK, JNK and p38 dependent pathways through binding to its receptors TGF-β-RI and TGF-β-RII [34]. To determine the pathways involved in the TGF-β1 dependent downregulation of RECK, NIH3T3 fibroblasts were treated with different specific inhibitors against the kinase activity of TGF-β-RI, Smad-3, PI3K, MEK-1 MAPK, JNK and p38, or treated with siRNAs against Smad-2 and -3, prior to TGF-β1 treatment. Fig 2A shows that the TGF-β1dependent downregulation of RECK requires the activation of TGF-β-RI, since the effect was inhibited when the cells were pre-treated with SB525334, a specific inhibitor TGF-β-RI kinase activity [35]. FN expression is showed as a control for the cellular response to TGF-β1, where the TGF-β1 dependent upregulation of FN was also inhibited by SB525334. Fig 2A also shows that LY294002, PD98059 or SB203580, specific inhibitors for the PI3K, MEK1, and p38 pathways respectively, did not interfere with TGF-β1 dependent RECK downregulation. However, pre-treatment with SB600125, a specific inhibitor of the JNK pathway, significantly reduces the effect of TGF-β1 over RECK levels, suggesting that TGF-β1 decreased RECK expression, at least in part, by a JNK dependent pathway. The inhibitors used in Fig 2A did not show any effect on RECK or FN expression levels (S1 Fig).

To evaluate the role of the TGF-β1 canonical pathway, NIH3T3 fibroblasts were transiently transfected with a siRNAs against Smad-2 and Smad-3, or treated with SIS3, a specific inhibitor of Smad-3 activation [36, 37]. Fig 2B shows that in fibroblasts transfected with siRNAs against Smad-2 and Smad-3, the negative effect of TGF-β1 over RECK expression was reduced by approximately 50%, compared to the scrambled siRNA transfected fibroblasts. The effect of the siRNA transfection was confirmed by observing decreased levels of Smad-3 protein by Western blot (Fig 2B). We also evaluated if decreasing the activation of Smad-3 by SIS3 treatment affects the TGF-β1 dependent downregulation of RECK. The Fig 2C shows that in fibroblasts pre-treated with SIS3, the TGF-β1 dependent downregulation of RECK was diminished. These results indicate that the canonical TGF-β1 pathway is required for the TGF-β1 dependent reduction of RECK expression. Fig 2C also shows that the inhibitory effect of SIS3 or JNK alone are comparable to the inhibitory effect obtained with both inhibitors in combination. These results indicate that both the canonical (Smad) and the non-canonical (JNK) TGF-β1 dependent pathways act in concert downregulating RECK expression in fibroblasts.

### TGF-β1 dependent up-regulation of β1-integrin expression require the concomitant reduction of RECK expression

**Fig 3:**
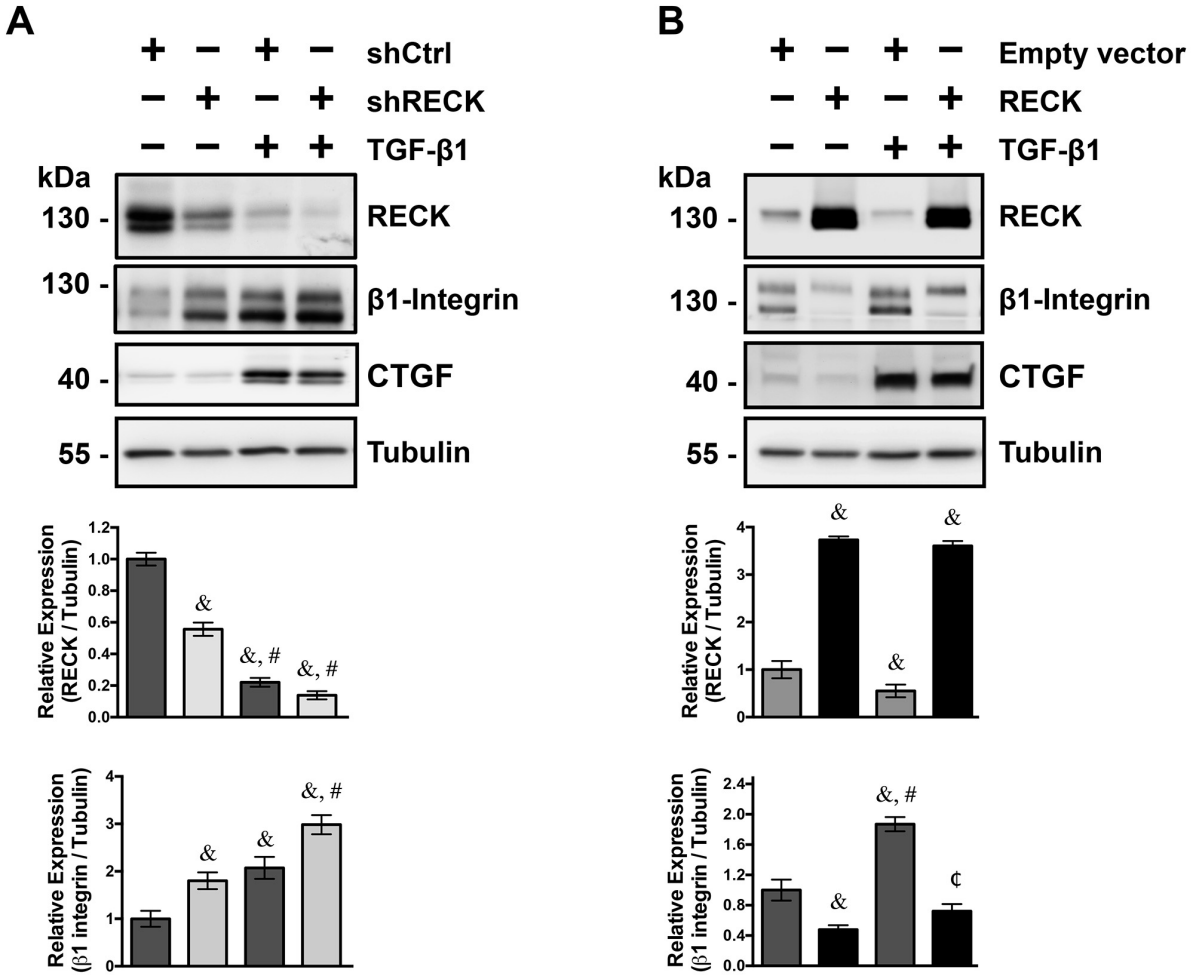
TGF-β1 must reduce RECK levels to increase β1-integrin levels in fibroblasts. **(A)** NIH3T3 fibroblasts were transiently transfected with a pool of shRNAs against mouse RECK (shRECK) or with a scrambled sequence as control (shCtrl). 24 h post-transfection, cells were treated or not with 5 ng/ml of TGF-β1 for 24 h (**B**) NIH3T3 fibroblasts were transiently transfected with a vector that overexpresses RECK or with an empty vector as control. 24 h post-transfection, cells were treated with 5 ng/ml of TGF-β1 for 24 h, or left untreated as a control. In A and B, Western blot analysis of cell extracts were performed to determine the levels of RECK, β1-integrin and CTGF. Tubulin levels were used as a loading control. In A, &, P<0.05 relative to untreated shCtrl; #, P<0.05 relative to untreated shRECK. In B, &, P<0.05 relative to TGF-β1 untreated Empty vector; #, P<0.05 relative to TGF-β1 untreated RECK; ¢, P<0.05 relative to TGF-β1 treated Empty vector.

To determine if β1-integrin expression and its regulation by TGF-β1 depends on RECK levels in fibroblasts, we transiently transfected NIH3T3 fibroblasts with a pool of shRNAs against RECK (shRECK) or a scrambled sequence (shCtrl) as control; 24 hours later the cells were treated with TGF-β1 for another 24 hours. The shRECK transfections decreased RECK levels up to a 60% of shCtrl-transfected fibroblasts (Fig 3A). Under these conditions, the expression of β1-integrin was induced almost twofold of shCtrl levels and to comparable levels of control TGF-β1 treated cells, evaluated by Western blot (Fig 3A). TGF-β1 treatment reduced RECK levels of both shCtrl and shRECK transfected fibroblasts, inducing, at the same time, the expression of β1-integrin. Interestingly the TGF-β1 dependent induction of β1-integrin expression was significantly higher in the fibroblasts transfected with the shRECK compared to control treated fibroblasts. Reduced RECK levels by shRECK transfection do not show any effect on the basal or TGF-β1 dependent induction of CTGF expression, suggesting that RECK levels have a specific effect over β1-integrin expression but not to other TGF-β1 regulated proteins.

In accordance with the above results, it is possible to hypothesize that the TGF-β1 dependent induction of β1-integrin expression depend on RECK downregulation. To evaluate this, we decided to overexpress the human version of RECK in fibroblasts, analyzing its effect on β1-integrin expression in response to TGF-β1. Since the overexpressed *Reck* is under the control of a strong, foreign promoter (SV40 promoter), its expression should not be affected by TGF-β1. Fig 3B shows that in sham-transfected fibroblasts (empty vector), TGF-β1 reduced RECK expression and at the same time induced β1-integrin expression. However, when RECK was overexpressed, the expression of β1-integrin levels were greatly reduced. In the RECK-overexpressing fibroblasts, we observed that RECK levels were unaltered by TGF-β1 treatment, while the upregulation of β1-integrin was greatly affected. RECK overexpression do not show any effect on the basal or TGF-β1 dependent induction of CTGF expression, suggesting that the cellular response to TGF-β1 was not hampered. The later conclusion was supported with a TGF-β1 reporter assay (p3TP-Lux [38]) as shown in the S2 Fig, indicating that the effect of RECK over β1-integrin expression is independent of the cellular response to TGF-β1.

All of these results strongly suggest that RECK expression levels are inversely correlated to β1-integrin levels and that the TGF-β1 dependent downregulation of RECK is a pre-requisite for the induction of β1-integrin in response to TGF-β1 in fibroblasts.

### Downregulation of RECK in fibroblasts is required for TGF-β1-induced matrix contraction

**Fig 4:**
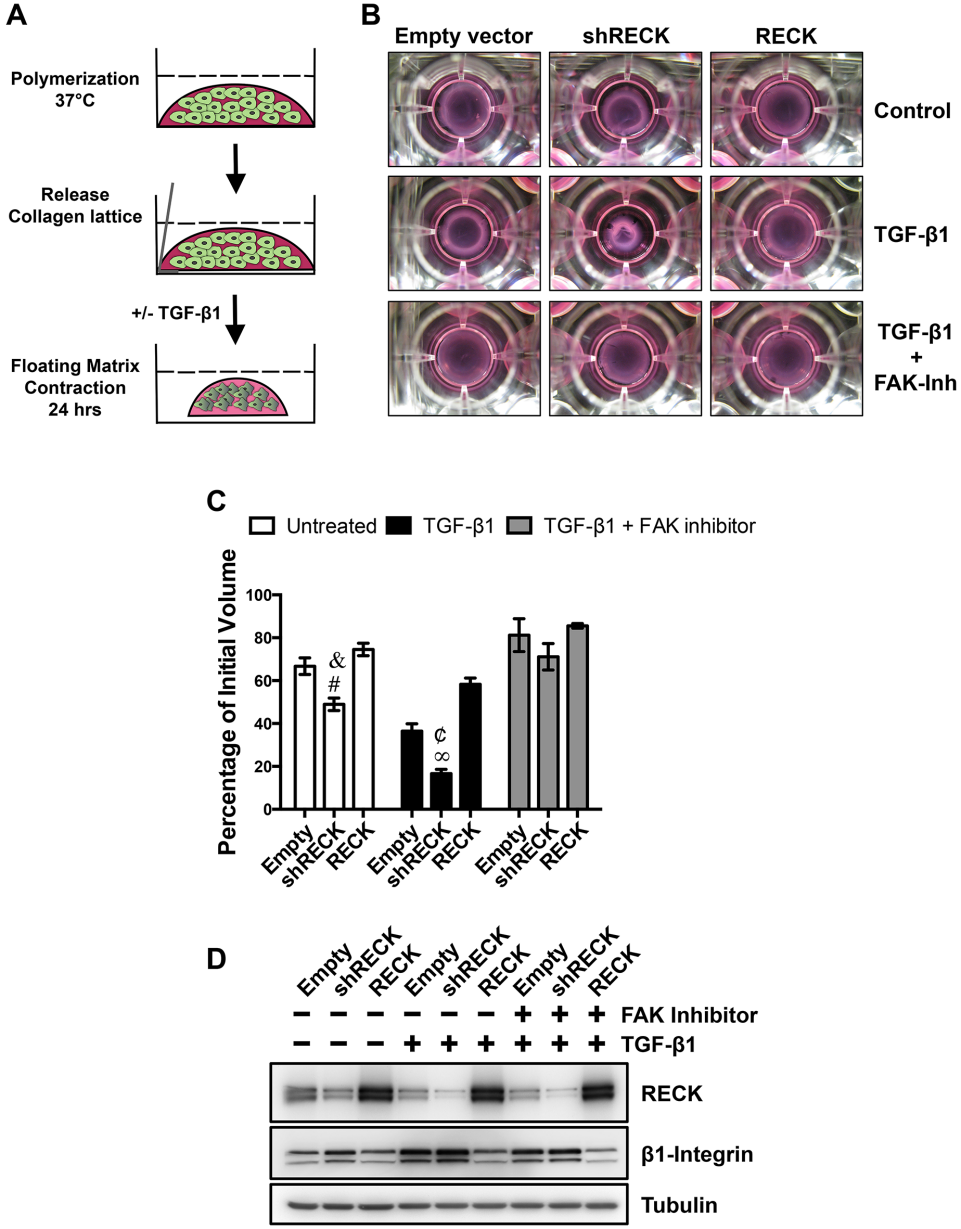
Downregulation of RECK in fibroblasts is required for TGF-β1 induced matrix contraction. **(A)** Schematic of the matrix contraction assay. From top to bottom, fibroblasts were mixed with a collagen solution that polymerizes into a 3D collagen matrix for 2 h. This mixture was then released from the culture dish and allowed to float in culture medium. Floating collagen lattices were incubated with TGF-β1 for 24 hours to induce fibroblasts contraction, leading to a volume reduction of the collagen lattice. **(B)** NIH3T3 fibroblasts were transiently transfected with a pool of shRNAs against mouse RECK (shReck) or with a RECK overexpression vector or with an empty vector as control. 24 h post-transfection, fibroblasts were trypsinized and a collagen contraction assay was performed as described in (A). After 24 h, the now trypsinized collagen lattices were treated with TGF-β1 and/or the focal adhesion kinase inhibitor 14 (FAK-Inh) for 24 h. Afterwards, 3D floating matrices were photographed. Representative images are shown. **(C)** The volume of the contracted matrices obtained in (B) was measured immediately after being released at the end of the assay. These values, obtained from two independent experiments performed in triplicate, were graphed as a percentage of the initial volume. Statistical significance was assessed using two-way ANOVA and a Bonferroni multiple-comparison post hoc test. &, P<0.05 relative to TGF-β1 untreated Empty vector; #, P<0.05 relative to TGF-β1 untreated RECK; ¢, P<0.05 relative to TGF-β1 treated Empty vector; ∞, P<0.05 relative to TGF-β1 treated RECK. **(D)** Aliquots of the trypsinized cells in (B) were directly seeded in culture dishes before mixing with the collagen solution and then treated with TGF-β1 and/or the FAK-Inh for 24 has described in (B). Cell extracts were prepared and analyzed by Western blot to determine the levels of RECK and β1-integrin. Tubulin levels were used as a loading control.

Since the mechanical forces executed by fibroblasts upon the ECM depend on integrins expression, we decided to study the role of RECK on fibroblast dependent ECM contraction. Fig 4A shows a schematic summary of the assay. Freshly polymerized 3D collagen matrices containing NIH3T3 fibroblasts were released from the bottom of the culture dish and allowed to float in the culture medium. The floating 3D collagen lattice was incubated in the presence of TGF-β1 for 24 hours to induce fibroblast contraction and therefore a volume reduction of the collagen lattice. S3 Fig shows the concentration dependent effect of TGF-β1 on fibroblasts in the collagen lattice contraction assay, as previously described [15, 39]. To study the role of RECK in this process, we transiently transfected NIH3T3 fibroblasts with shRECK or with the RECK expression vector or an empty vector as control. Twenty-four hours post transfection, the fibroblasts were trypsinized, mixed with collagen, and allowed to polymerize. Afterwards, the floating collagen embedded cells were left untreated as control or incubated with TGF-β1 for 24 hours. Fig 4B shows representative photographs of the floating collagen lattices at the end of the assay. The volume of these collagen lattices was determined and expressed as the percentage of the initial volume, as shown in Fig 4C. In the absence of TGF-β1, all collagen lattices showed a basal contraction. Control and RECK overexpressing fibroblasts reduced their collagen lattice volumes by around 30%. This contraction was more pronounced in the shRECK fibroblasts, reaching a 50% reduction of the initial volume. TGF-β1 treatment reduces the volume of the matrices by 65% in control fibroblasts and by 80% in the RECK deficient fibrobla*s*ts (shRECK). Conversely, RECK overexpression inhibits the TGF-β1 dependent contraction. In this case, the volume reduction was comparable to the untreated control fibroblasts.

Taking into account that the integrin dependent ECM contraction requires the activation of FAK, we decided to chemically inhibit this activation, to evaluate whether the increased amount of β1-integrin observed in shRECK fibroblasts, explains the enhanced contraction. To this, we treated the WT, shRECK, and RECK transfected fibroblasts with TGF-β1 in the presence of a FAK inhibitor (FAK Inhibitor). As shown in the bottom panels of Fig 4B and in the quantification in Fig 4C, the presence of the FAK inhibitor inhibited the contraction in all cases, indicating the requirement of FAK activity. Thus the induced integrin expression and its canonical signaling explain the enhanced ECM contraction observed in RECK deficient fibroblasts. As control, we determined the expression levels of RECK and β1-integrin in this assay, as shown in Fig 4D. The presence of FAK inhibitor in TGF-β1 treated cells did not affect the levels of RECK or β1-integrin as compared to the TGF-β1 treated fibroblasts.

**Fig 5:**
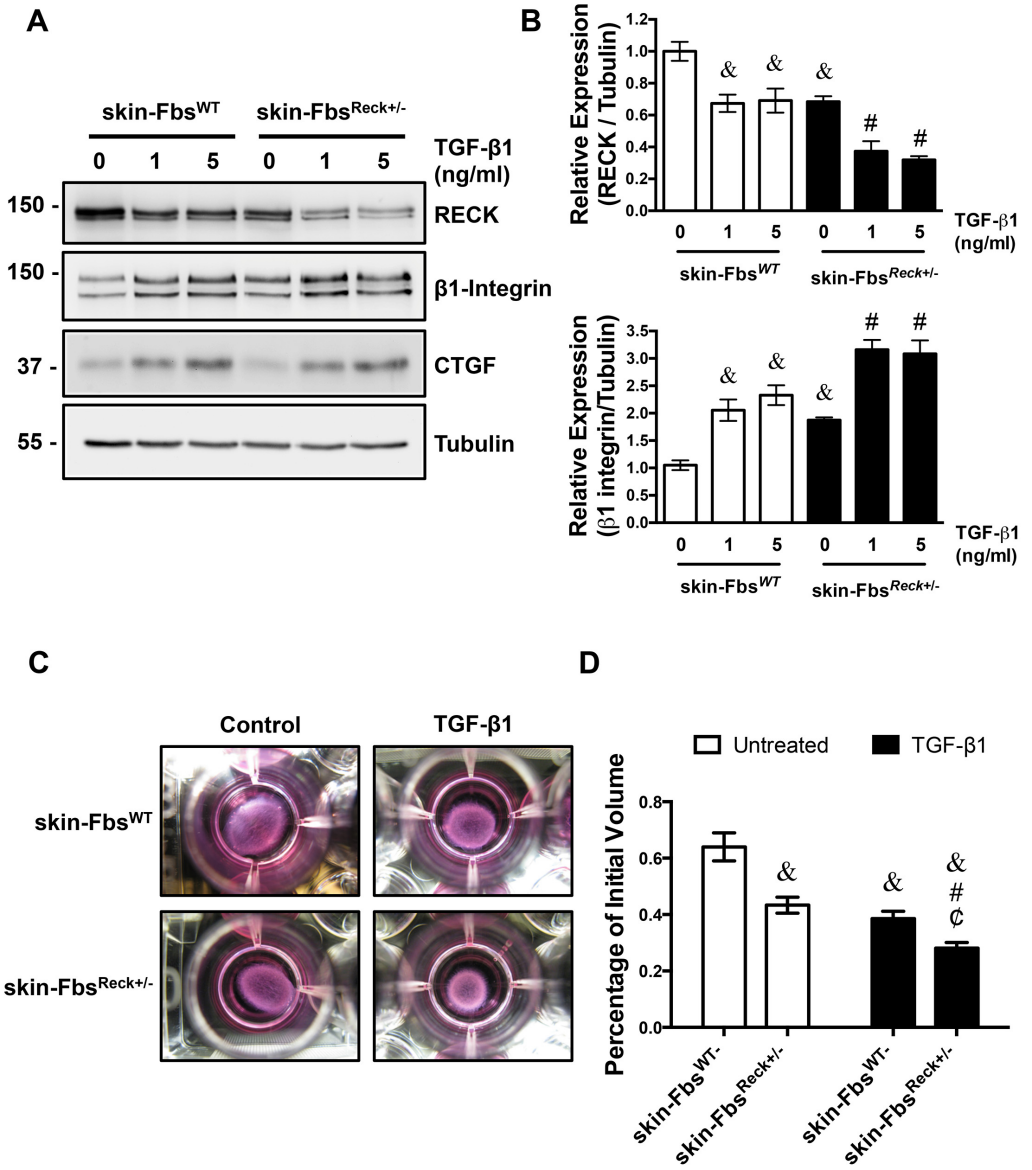
*Reck* ^+/-^ fibroblasts show increased level of β1-integrin and enhanced contractile properties. **(A)** Primary fibroblasts cultures derived from skin biopsies of 3 month old WT (skin-Fbs^WT^) and *Reck* ^*+/-*^ (skin-Fbs^Reck+/-^) mice were incubated with TGF-β1 for 24 hours as indicated. Western blot analysis of cell extracts were performed to determine the levels of RECK, β1-integrin and CTGF. Tubulin levels were used as a loading control. **(B)** Quantification of the expression of RECK and β1-integrin relative to tubulin expression. values are expressed as mean +/- SD of two independent experiments. &, P<0.05 relative to TGF-β1 untreated skin-Fbs^WT^; #, P<0.05 relative to TGF-β1 untreated skin-Fbs^Reck+/-^. **(C)** A collagen contraction assay using primary culture fibroblasts derived from skin biopsies of 3 month old WT (skin-Fbs^WT^) and *Reck* ^*+/-*^ (skin-Fbs^Reck+/-^) mice was performed. The floating collagen lattices were treated with TGF-β1 (3 ng/ml). After 24 hours of treatment, the 3D floating matrices were photographed. Representative images are shown. **(D)** The volumes of the contracted matrices obtained in (A) were measured immediately after being released at the end of the assay. The values, obtained from two independent experiments performed in triplicate, were graphed as a percentage of the initial volume. Statistical significance was assessed using two-way ANOVA and a Bonferroni multiple-comparison post hoc test. &, P<0.05 relative to TGF-β1 untreated skin-Fbs^WT^; #, P<0.05 relative to TGF-β1 untreated skin-Fbs^Reck+/^; ¢, P<0.05 relative to TGF-β1 treated skin-Fbs^WT^.

In order to evaluate if the observed results are reproduced in primary mouse fibroblasts cultures, we used primary cultures of skin fibroblasts from the hemizygous *Reck* mice (skin-Fbs^Reck+/-^) and from WT mice (skin-Fbs^WT^). Fig 5A shows that the expression of RECK was reduced by almost 50% in the skin-Fbs^Reck+/-^ compared skin-Fbs^WT^ (Fig 5B). In addition, we observed that in both cases, TGF-β1 treatment reduced RECK expression. Agreeing with our previous results, the expression of β1-integrin was higher in the skin-Fbs^Reck+/-^ compared to skin-Fbs^WT^, and this difference was exacerbated upon TGF-β1 treatment (Fig 5B). Meanwhile, the expression of CTGF, evaluated as a control of the cellular response to TGF-β1, was identical in both skin-Fbs^WT^ and skin-Fbs^Reck+/-^.

We also evaluate the contraction capacity of skin-Fbs^WT^ compared to skin-Fbs^Reck+/-^. Fig 5C shows representative photographs of the collagen lattices at the end of the assay. The contraction percentages relatives to the initial volume of the collagen lattices are indicated in Fig 5D. Both skin-Fbs^WT^ and skin-Fbs^Reck+/-^ exhibited matrix contraction under basal conditions, which were augmented upon TGF-β1 treatment. In absence of TGF-β1, skin-Fbs^WT^ showed 36% volume reduction after 24h. The skin-Fbs^Reck+/-^ showed more evident contraction, 58% of volume reduction. This was comparable to the volume of the TGF-β1 treated skin-Fbs^WT^, which had 62% volume reduction. The treatment of skin-Fbs^Reck+/-^ with TGF-β1 reduced the matrix volume by 72%. Altogether, these results indicate that the integrin-FAK dependent fibroblast contraction induced by TGF-β1 is required and enhanced by reduced RECK levels.

### *Reck* deficient mice show accelerated skin wound contraction

**Fig 6:**
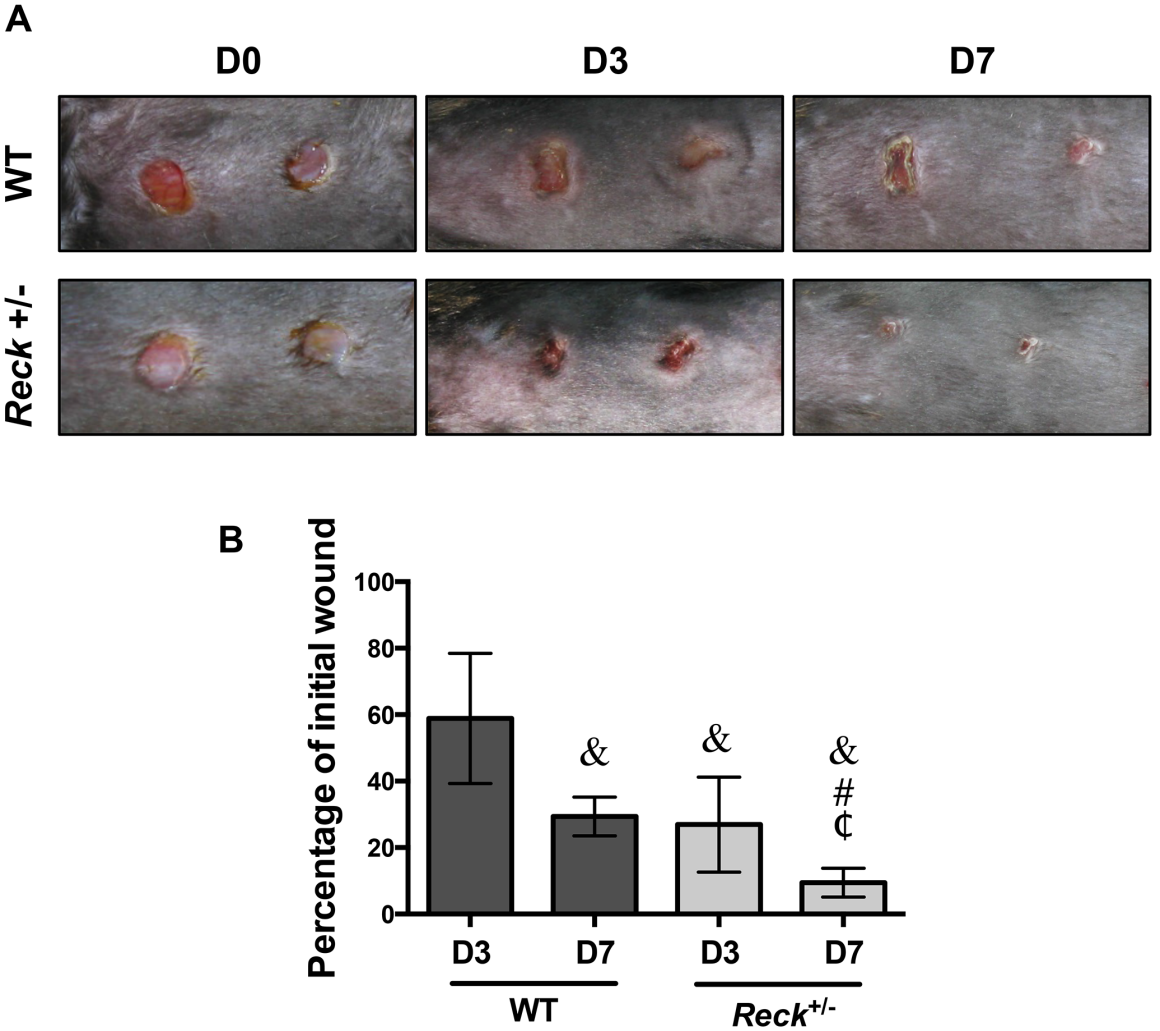
*Reck* deficient mice have accelerated skin wound contraction. A dermal punch wound contraction assay on the back of 3- month-old WT and *Reck* ^*+/-*^ mice was performed. The wounds were photographed at the moment of the surgery (D0) and after 3 (D3) and 7 (D7) days. Representative photographs are shown in **(A)**. The wounded area was determined at D0, D3 and D7 and expressed as percentage of the initial wound area, as shown in **(B)**. Values correspond to mean ± S.D. of two independent experiments, using four mice for each experimental condition. Statistical significance was assessed using two-way ANOVA and a Bonferroni multiple-comparison post hoc test. &, P<0.05 relative to WT D3; #, P<0.05 relative to WT D7; ¢, P<0.05 relative *RECK+/-* D3.

Next we decided to evaluate if the process of skin wound contraction is enhanced in *Reck* ^*+/-*^ mice skin. We performed an *in vivo* wound contraction assay on 3-month-old mice through dermal punch wounding. Fig 6A shows representative photographs of the performed wounds at the day of the surgery (D0), or after 3 (D3) and 7 (D7) days from the surgery. The wounded area was determined at D0, D3 and D7 and expressed as a percentage of the initial wounded area as shown in Fig 6B. Notably, *Reck*+/- mice showed a higher rate of wound closure at 3 and 7 days after injury when compared with WT injured mice at the same days after injury. At D3, wounds in WT animals closed to 60% of the initial wounded area, which is consistent with previous observations [40–42]. Interestingly, at the same time the cutaneous wounds in the *Reck*+/- mice closed to less than 30% of the initial wounded area, and were comparable to the wounded area in the WT animals after 7 days. At D7, the wounds of the *Reck*+/- mice were almost totally closed, to only 11% of the initial wounded area. These results strongly suggest that RECK play an essential role for proper wound closure.

## Discussion

In this paper, we have demonstrated in fibroblasts that TGF-β1 downregulates the expression levels of RECK through Smad and JNK dependent pathways, which is concomitant to the increase of β1-integrin expression. The latter is simultaneously dependent on the downregulation of RECK. In agreement with these findings, we showed that fibroblasts with reduced expression of RECK, by the transient transfection of shRNA against mouse RECK or obtained from *Reck* ^+/-^ mice, express more β1-integrin and showed increased contractile properties compared to wild type fibroblasts, in both cases potentiated by TGF-β1 treatment. Notably, we observed that *Reck* ^+/-^ mice close dermal wounds more efficiently than wild type mice, suggesting an unexpected role for RECK in wound contraction.

Here we show that in fibroblasts TGF-β1 mediates the downregulation of both RECK mRNA and protein levels at the same time, suggesting that the regulation is at the mRNA transcriptional level. The TGF-β1 dependent downregulation of RECK protein levels requires the activation of the Smad and JNK pathways. Inhibition of p38, ERK1/2, or PI3K did not show any effect on RECK protein levels. However it cannot be discarded that RECK decrease observed in our experiments is a consequence of an inhibition on its synthesis and/or an increase on its degradation.

It has been previously described that RECK expression levels can be regulated by TGF-β1 in different cancer derives cell lines [43, 44] and during the activation of pancreatic stellate cells (PSCs) [45]. In all these cases, the mechanism and signaling pathway involved are different, indicating the complex regulation of RECK expression by TGF-β1. In highly invasive breasts cancer cells (MDA-MB-231), TGF-β1-treatment increased the expression of RECK at the mRNA level but diminished the RECK protein levels p38 but not ERK1/2 dependent pathways inhibition, blocked the TGF-β1-mediated increase of RECK mRNA expression. However, ERK1/2 inhibition, but not p38 inhibition, blocked the TGF-β1-mediated downregulation of RECK protein levels. This indicates that TGF-β1 have opposite effects over RECK mRNA and protein levels in breast cancer cells [43]. Moreover, TGF-β1 treatment of Panc-1 cells, a pancreatic cancer cell line, led to a twofold increase in RECK protein levels without any effect on RECK mRNA levels, which was dependent on the activation of the Smad dependent pathway [44]. Otherwise, the treatment of activated PSC with TGF-β1 induces an increase in the protein levels of RECK without changing the RECK mRNA levels. Here the evidences indicate that TGF-β1 through the Smad dependent pathway protects RECK from proteolytic degradation [45].

Recently it has been described that the TGF-β1-induced epithelial-mesenchymal transition in non-tumorigenic epithelial cells, defined by E-cadherin downregulation, is accompanied by RECK upregulation by a mechanism that involve beta catenin. However, the loss of E-cadherin expression by TGF-β1 treatment is uncoupled from RECK upregulation in different carcinoma-derived cell lines. In fact TGF-β1 failed to upregulate, or even downregulate, RECK levels in these cell lines. All of these demonstrate the highly complex regulation of RECK [46].

We show that decreased RECK expression levels in *Reck* ^+/-^ mice are associated with increased wound contraction efficiency when compared to wild type mice. The inverse relation that was observed between β1-integrin and RECK expression levels can explain this. Fibroblasts with low expression levels of RECK showed increased levels of β1-integrin. This was associated with an increased capacity for matrix contraction when evaluated using the floating collagen lattice assay. All of these observations were potentiated by TGF-β1 treatment and dependent on FAK activation. Previously, a relation has been reported between RECK and the role of FAK on the behavior of fibroblasts [47]. Fibroblasts lacking RECK expression showed decreased spreading, altered anterior–posterior polarity, increased speed, decreased directional persistence in migration, increased levels of activated Rac1 and Cdc42, and stabilized microtubules when cultured on plastic. These effects may be explained by the inability of the RECK deficient cells to form discrete focal adhesions. However, this phenotype is largely suppressed in the presence of FN or collagen I as substrates in the culture dishes [29]. Here we show that in fibroblasts cell lines and also in primary cultures of mouse fibroblasts derived from different tissues obtained of the WT and *Reck* ^+/-^ mice, RECK expression are inversely related to β1-integrin expression levels. Moreover, TGF-β1 induced the downregulation of RECK expression, which was found to be necessary for the TGF-β1 dependent upregulation of β1-integrin. This occurred concomitant to the TGF-β1 dependent induction of FN expression, which accumulates in the ECM. We also observed that the induced expression of β1-integrin in RECK deficient fibroblasts was associated with a higher capacity for contraction compared to WT fibroblasts. This contraction was enhanced by TGF-β1 stimulation. It should be noted that in this assay, fibroblasts were embedded in a collagen matrix. Consequently, in these ECM-enriched scenarios, the behavior of fibroblasts probably favors attachment due to the increased levels of β1-integrin. Nevertheless, these data clearly show that the lack of RECK has a significant impact on the behavior of fibroblasts.

New evidences are required to clarify how RECK levels affect β1-integrin expression levels; for instance, it has been described that RECK regulates β1-integrin activity and downstream dependent signaling [28, 29]. In human endothelial cells, RECK depletion is related to decreased β1-integrin activation and diminished FAK activity, which has previously been associated with decreased proliferation and a defective capacity to form vascular tubes [28]. Although there has previously been no evidence for the direct role of RECK in signaling that could affects β1-integrin levels, this possibility cannot be discarded. For example, RECK appears to regulate the activation of Notch signaling [48–50] and as Notch signaling is involved in different regeneration processes [51], this regulation could signify a potential mechanism for β1-integrin regulation. In addition, we cannot exclude the possibility that MMPs regulated by RECK: MMP-2, MMP-9 and MT1-MMP could also be participating in this mechanism. Indeed, some reports have shown the participation of MMPs, specifically MT1-MMP, in the regulation of signaling pathways [52].

Our main contribution in this study was to demonstrate a critical role for RECK in fibroblasts contracting capabilities, revealing a new pathway regulating wound contraction: the axis TGF-β1—RECK- β1-integrin. This was particularly interesting since traditionally, RECK has been associated with pathological processes, specifically cancer where the expression of RECK has been observed to be reduced in several types of tumors. This reduction has consequently been associated to an increase in the invasiveness of malignant cancer cells arising from increased metalloproteinase activity [53–55]. However, in the case of wound healing, the reduction of RECK levels seems to be critical for proper wound closure, as we demonstrated using Reck^+/-^ mice, where reduced RECK levels were associated with increased wound closure speed.

Finally, this study opens the door to new investigations and proposes RECK as a novel target that can be used for the development of new therapies associated with regenerative medicine.

## Methods

### Cell culture

The murine embryonic fibroblasts cell lines NIH3T3 were obtained from American Type Culture Collection (ATCC) (Rockville, MD, USA) and grown as previously described [10].

Primary fibroblasts cultures from skeletal muscle were obtained from tibialis anterior muscle biopsies from 3-month-old C57/Bl6, as described previously [56]. Skin, primary fibroblast cultures, was obtained from the skin biopsies generated by the wound contraction assay (see below) as previously described [57], with some modifications. Briefly, the skin biopsies obtained were minced into small pieces, seeded onto 3.8 cm^2^ gelatin coated well plates and covered with 500 μL of growth medium (Dulbecco’s modified Eagle’s medium F-12, 100 U/ml penicillin, 0.1 mg/ml streptomycin and 0.25 μg/ml amphotericin B, Invitrogen) supplemented with 50% (v/v) fetal bovine serum (Hyclone). Explants were kept at 37°C, 5% CO_2_ and 95% humidity. After 3–4 days, fibroblasts migrated from the muscle explants. When the cells covered 60–70% of the well surface, the cells were trypsinized and plated onto 21 cm^2^ petri dishes and growth medium was switched to 20% FCS. Cells were used in passages 2 to 4. All protocols were conducted in strict accordance and with the formal approval of the animal Ethics Committee of the Pontificia Universidad Católica de Chile.

Cells were treated with TGF-β1 (R&D, Minneapolis, MN, USA) in Dulbecco’s modified Eagle’s medium supplemented with 2% (v/v) fetal bovine serum and penicillin/streptomycin in a 5% CO_2_ atmosphere at the concentration and time indicated in the corresponding figure legend. The following inhibitors were added to the cell media 30 minutes prior to TGF-β1 treatment: (ALK5)/ type I TGF-β-receptor kinase inhibitor SB525334 and the focal adhesion kinase (FAK) inhibitor, FAK inhibitor 14, from Tocris Biosciences, (San Diego CA, USA), the PI-3 kinase inhibitor LY294001 from Cambridge Bioscience Ltd (Cambridge, UK), the inhibitor of MAPK/ERK kinases PD98059, the inhibitor of p38 MAPK phosphorylation SB203580, the inhibitor of JNK activity SB600125 were from Cell Signaling (Danvers, MA, USA) and the Smad-3 phosphorylation inhibitor SIS3 from Millipore, USA)

### Transfections

The empty expression vector plxsb, the expression vector containing the sequence of human RECK [55] and the MISSION TRC shRNA against mouse RECK were kindly donated by Chiaki Takahashi. Transfections were carried out using Lipofectamine 2000 (Invitrogen, USA), according to the supplier’s protocol. Cells were treated with TGF-β1 24 hours after transfection and evaluated at the indicated times.

### Floating collagen-based contraction assays and quantitation of gel contraction

Primary skin fibroblasts or NIH3T3 fibroblasts (24 hours after transfection as indicated in each case) were subjected to a collagen-based contraction analysis using the Cell Contraction Assay kit (Cell Biolabs, Inc. San Diego, CA USA) according to the supplier’s protocol. Briefly, trypsinized fibroblasts were suspended in DMEM F12 supplemented with 5% FBS at a density of 5 X 10^6^ cells per ml. The collagen lattices were prepared by mixing 1 part of the cell suspension (60 μl) with 4 parts of the collagen solution (prepared with the reagents supplied with the kit) yielding a final concentration of 1 X 10^6^ cells per ml and 1.85 mg per ml of bovine type I collagen. A volume of 300μl of the collagen/cell suspension was added to the required wells of a 24-well tissue culture plate. The plates were incubated at 37°C, 5% CO_2_ and 95% humidity for 2 hours. After polymerization, 1 ml of DMEM F12 supplemented with 1% FBS with or without 1 to 5 ng/ml of TGF-β1 were added to each well. Collagen lattices were photographed 24 hours afterwards. The volume of the contracted matrices was measured at the beginning and end of the assay. The values were graphed as a percentage of the initial volume.

### Animal procedures

C57BL/6 mice carrying a hemizygous mutation in the RECK gene were generously provided by Chiaki Takahashi [26]. All mice had free access to water and food until they were studied at a 3-month time point. At the end of the experiments, the mice were anaesthetized by isofluorane gas and euthanized by cervical dislocation. All protocols were conducted in strict accordance and with the formal approval of the Animal Ethics Committee of the Pontificia Universidad Católica de Chile and Fondo Nacional de Desarrollo Cientifico y Tecnologico (FONDECYT Grant number 11110010, Chile).

### Wound contraction assays

Analysis of wound repair was performed in 3-month-old mice. When performing skin excision, the mice were anaesthetized by isofluorane gas, shaved, and two 3 mm diameter skin biopsies were taken with a sterile disposable biopsy punch, as described previously [41], immediately after wounding, an aliquot of povidone iodine solution (7,5%) was topically applied to avoid possible infections. The wound area was allowed to heal in the open air and photographs were taken at the indicated times. Data were plotted as a percentage of the open area.

### RNA isolation, reverse transcription and quantitative PCR

Total RNA containing microRNAs was isolated from cell cultures using mirVana miRNA Isolation Kit according to the manufacturer’s instructions (Life Technologies, USA). Reverse transcription into complementary DNA was performed using SuperScript III Reverse Transcriptase and random hexamers according to the manufacturer’s instructions (Life Technologies, USA). Taqman quantitative real-time PCRs were performed in triplicate on an Eco Real-Time PCR System (Illumina, USA) using pre-designed primer sets for mouse RECK, mouse β1-integrin, and the housekeeping gene GAPDH (Taqman Assays-on-Demand, Applied Biosystems, USA). mRNA expression was quantified using the comparative ΔC_T_ method (2-ΔΔC_T_) with GAPDH as the reference gene. The mRNA levels were expressed relative to the mean expression of the control, untreated cells.

### Cell extracts, SDS-PAGE, and western blot

Cell extracts were prepared in lysis buffer: 50mM Tris, pH 7.4, 100 mM NaCl, 0.5% Triton X-100 and 1% SDS supplemented with a Halt Protease & Phosphatase Inhibitor Cocktail (Pierce, USA). To analyze phosphorylated proteins, cell extracts were prepared in RIPA buffer in the presence of phosphatase inhibitors as previously described [58]. Cell extracts were also obtained from the collagen lattices. To accomplish this, the gels were incubated in 0.2% collagenase I (Sigma-Aldrich) for 3 hours. 2X lysis buffer was added to aliquots of the digestion solutions. Protein content was determined by the tubulin content observed through western blot. Aliquots with equivalent amounts of protein were subjected to SDS-PAGE in 8% polyacrylamide gels, electrophoretically transferred to Immobilon membranes (Millipore, Bedford, MA, USA) and probed with the following: rabbit anti-RECK (Cell signaling, 1/1000); rabbit anti-fibronectin (1/2000, Sigma-Aldrich); goat anti-CTGF (Santa cruz biotechnology, 1/500); mouse anti-myogenin (Santa cruz biotechnology, 1/500); mouse anti-β1 integrin (B&D transduction laboratories, 1/1000); rabbit anti-TGF-β-Receptor I (Santa cruz biotechnology, 1/300); rabbit anti-TGF-β-Receptor II (Cell signaling, 1/500), rabbit anti-phospho Smad-2 (Cell signaling, 1/1000); anti-total Smad-2 (Cell signaling, 1/1000), rabbit anti-phospho Smad-3 (Cell signaling, 1/1000); rabbit anti-total Smad-3 (Cell signaling, 1/1000) and mouse anti-β-tubulin (1/5000) (Sigma-Aldrich). All immunoreactions were visualized by enhanced chemiluminescence (Pierce, Rockford, IL, USA), using a ChemiDoc-It HR 410 imaging system (Upland, CA, USA).

### Protein quantification

Protein content in cell extracts was determined with the bicinchoninic acid protein assay kit (Pierce) with BSA as a standard, according to the manufacturer’s protocol.

### Statistical analyses

The number of replicates used for each experiment is indicated in their respective figure legends. Data are presented as the mean ± standard deviation. Statistical significance was assessed using two-way ANOVA and a Bonferroni multiple-comparison post-hoc test. Differences were considered statistically significant with a p value < 0.05.

## Supporting Information

S1 Fig TGF-β1 decreases RECK levels through Smad and JNK dependent pathways. **(A)** NIH3T3 fibroblasts were pre-treated for 30 minutes with different inhibitors: TGF-β-RI kinase inhibitor SB525334, PI3K inhibitor LY294002, MEK1 inhibitor PD98059, JNK inhibitor SB600125 and p38 inhibitor SB203580. After the pre-treatment, fibroblasts were treated with 5 ng/ml of TGF-β1 for 24 hours or left untreated as a control. Western blot analysis of cell extracts were performed to determine the levels of RECK and FN. β-tubulin levels were used as a loading control. **(B)** NIH3T3 fibroblasts were pre-treated for 30 minutes with SIS3, a specific inhibitor of Smad-3 activation, and the JNK inhibitor SB600125; fibroblasts were treated with either inhibitor alone or in combination. Cells were then treated with 5 ng/ml of TGF-β1 for 24 hours, or left untreated as a control. Western blot analysis of cell extracts were performed to determine the levels of RECK and FN. Tubulin levels were used as a loading control. The quantifications shown in A and B are derived from two independent experiments. Statistical significance was assessed using two-way ANOVA and a Bonferroni multiple-comparison post hoc test. &, P<0.05 relative to TGF-β1 untreated fibroblasts.
(TIF)

S2 Fig Fibroblast response to TGF-β1 is independent of the expression levels of Reck. NIH3T3 fibroblasts were transiently co-transfected with the TGF-β1 reporter system p3TPLux/pRL and: with shRECK, with a RECK overexpression vector or with an empty vector as control. 24 h post-transfection, the cells were incubated with TGF-β1 at the indicated concentrations. Luciferase activity was determined after 24 hours of TGF-β1 treatment. The quantifications is from two independent experiments. Statistical significance was assessed using two-way ANOVA and a Bonferroni multiple-comparison post hoc test. &, P<0.05 relative to 0 ng/ml TGF-β1; #, P<0.05 P<0.05 relative to 1 ng/ml TGF-β1 in each case.
(TIF)

S3 Fig TGF-β1 concentration influences fibroblast-induced matrix contraction. **(A)** NIH3T3 fibroblasts were subjected to a collagen contraction assay, as described in Fig 6A. After 24 hours of treatment with TGF-β1 at two different concentrations, as indicated, the 3D floating matrices were photographed. Representative images are shown. **(B)** The volume of the contracted matrices obtained was measured immediately after being released at the end of the assay and graphed as a percentage of the TGF-β1 untreated fibroblast volume.
(TIF)

## References

[1] Gurtner GC, Werner S, Barrandon Y, Longaker MT. Wound repair and regeneration. Nature. 2008;453(7193):314–21. Epub 2008/05/16. 10.1038/nature07039 . 18480812

[2] Singer AJ, Clark RA. Cutaneous wound healing. N Engl J Med. 1999;341(10):738–46. Epub 1999/09/02. 10.1056/NEJM199909023411006 . 10471461

[3] Bainbridge P. Wound healing and the role of fibroblasts. Journal of wound care. 2013;22(8):407–8, 10–12. Epub 2013/08/09. 10.12968/jowc.2013.22.8.407 . 23924840

[4] Li B, Wang JH. Fibroblasts and myofibroblasts in wound healing: force generation and measurement. Journal of tissue viability. 2011;20(4):108–20. Epub 2009/12/10. 10.1016/j.jtv.2009.11.004 19995679 PMC2891362

[5] Derynck R, Zhang YE. Smad-dependent and Smad-independent pathways in TGF-beta family signalling. Nature. 2003;425(6958):577–84. Epub 2003/10/10. 10.1038/nature02006 nature02006 [pii]. . 14534577

[6] Leask A. Potential therapeutic targets for cardiac fibrosis: TGFbeta, angiotensin, endothelin, CCN2, and PDGF, partners in fibroblast activation. Circ Res. 2010;106(11):1675–80. Epub 2010/06/12. 10.1161/CIRCRESAHA.110.217737 106/11/1675 [pii]. . 20538689

[7] Vial C, Zuniga LM, Cabello-Verrugio C, Canon P, Fadic R, Brandan E. Skeletal muscle cells express the profibrotic cytokine connective tissue growth factor (CTGF/CCN2), which induces their dedifferentiation. J Cell Physiol. 2008;215(2):410–21. Epub 2007/12/08. 10.1002/jcp.21324 . 18064627

[8] Pakyari M, Farrokhi A, Maharlooei MK, Ghahary A. Critical Role of Transforming Growth Factor Beta in Different Phases of Wound Healing. Adv Wound Care (New Rochelle). 2013;2(5):215–24. Epub 2014/02/15. 10.1089/wound.2012.0406 10.1089/wound.2012.0406 [pii]. 24527344 PMC3857353

[9] Denton CP, Khan K, Hoyles RK, Shiwen X, Leoni P, Chen Y, et al Inducible lineage-specific deletion of TbetaRII in fibroblasts defines a pivotal regulatory role during adult skin wound healing. J Invest Dermatol. 2009;129(1):194–204. Epub 2008/06/20. 10.1038/jid.2008.171 jid2008171 [pii]. . 18563179

[10] Droppelmann CA, Gutierrez J, Vial C, Brandan E. Matrix metalloproteinase-2-deficient fibroblasts exhibit an alteration in the fibrotic response to connective tissue growth factor/CCN2 because of an increase in the levels of endogenous fibronectin. J Biol Chem. 2009;284(20):13551–61. Epub 2009/03/12. 10.1074/jbc.M807352200 M807352200 [pii]. 19276073 PMC2679456

[11] Stawowy P, Margeta C, Kallisch H, Seidah NG, Chretien M, Fleck E, et al Regulation of matrix metalloproteinase MT1-MMP/MMP-2 in cardiac fibroblasts by TGF-beta1 involves furin-convertase. Cardiovasc Res. 2004;63(1):87–97. Epub 2004/06/15. 10.1016/j.cardiores.2004.03.010 S0008636304001269 [pii]. . 15194465

[12] Hadler-Olsen E, Fadnes B, Sylte I, Uhlin-Hansen L, Winberg JO. Regulation of matrix metalloproteinase activity in health and disease. FEBS J. 2011;278(1):28–45. Epub 2010/11/23. 10.1111/j.1742-4658.2010.07920.x . 21087458

[13] Thannickal VJ, Lee DY, White ES, Cui Z, Larios JM, Chacon R, et al Myofibroblast differentiation by transforming growth factor-beta1 is dependent on cell adhesion and integrin signaling via focal adhesion kinase. J Biol Chem. 2003;278(14):12384–9. Epub 2003/01/18. 10.1074/jbc.M208544200 M208544200 [pii]. . 12531888

[14] Cabello-Verrugio C, Santander C, Cofre C, Acuna MJ, Melo F, Brandan E. The internal region leucine-rich repeat 6 of decorin interacts with low density lipoprotein receptor-related protein-1, modulates transforming growth factor (TGF)-beta-dependent signaling, and inhibits TGF-beta-dependent fibrotic response in skeletal muscles. J Biol Chem. 2012;287(9):6773–87. Epub 2011/12/29. 10.1074/jbc.M111.312488 22203668 PMC3307262

[15] Liu S, Xu SW, Blumbach K, Eastwood M, Denton CP, Eckes B, et al Expression of integrin beta1 by fibroblasts is required for tissue repair in vivo. J Cell Sci. 2010;123(Pt 21):3674–82. Epub 2010/10/14. 10.1242/jcs.070672 jcs.070672 [pii]. . 20940256

[16] Singh P, Chen C, Pal-Ghosh S, Stepp MA, Sheppard D, Van De Water L. Loss of integrin alpha9beta1 results in defects in proliferation, causing poor re-epithelialization during cutaneous wound healing. J Invest Dermatol. 2009;129(1):217–28. Epub 2008/07/18. 10.1038/jid.2008.201 jid2008201 [pii]. 18633440 PMC3681306

[17] Hoffman LM, Jensen CC, Chaturvedi A, Yoshigi M, Beckerle MC. Stretch-induced actin remodeling requires targeting of zyxin to stress fibers and recruitment of actin regulators. Mol Biol Cell. 2012;23(10):1846–59. Epub 2012/03/30. 10.1091/mbc.E11-12-1057 22456508 PMC3350550

[18] Midwood KS, Mao Y, Hsia HC, Valenick LV, Schwarzbauer JE. Modulation of cell-fibronectin matrix interactions during tissue repair. The journal of investigative dermatology Symposium proceedings / the Society for Investigative Dermatology, Inc [and] European Society for Dermatological Research. 2006;11(1):73–8. Epub 2006/10/31. . 17069013 10.1038/sj.jidsymp.5650005

[19] Rustad KC, Wong VW, Gurtner GC. The role of focal adhesion complexes in fibroblast mechanotransduction during scar formation. Differentiation; research in biological diversity. 2013;86(3):87–91. Epub 2013/04/30. 10.1016/j.diff.2013.02.003 . 23623400

[20] Dogic D, Eckes B, Aumailley M. Extracellular matrix, integrins and focal adhesions. Current topics in pathology Ergebnisse der Pathologie. 1999;93:75–85. Epub 1999/05/26. . 10339900 10.1007/978-3-642-58456-5_8

[21] Leask A. Focal Adhesion Kinase: A Key Mediator of Transforming Growth Factor Beta Signaling in Fibroblasts. Adv Wound Care (New Rochelle). 2013;2(5):247–9. Epub 2014/02/15. 10.1089/wound.2012.0363 24527346 PMC3857352

[22] Liu S, Xu SW, Kennedy L, Pala D, Chen Y, Eastwood M, et al FAK is required for TGFbeta-induced JNK phosphorylation in fibroblasts: implications for acquisition of a matrix-remodeling phenotype. Mol Biol Cell. 2007;18(6):2169–78. Epub 2007/04/06. doi: E06-12-1121 [pii] 10.1091/mbc.E06-12-1121 17409352 PMC1877111

[23] Hinz B. Formation and function of the myofibroblast during tissue repair. J Invest Dermatol. 2007;127(3):526–37. Epub 2007/02/15. doi: 5700613 [pii] 10.1038/sj.jid.5700613 . 17299435

[24] Tomasek JJ, Gabbiani G, Hinz B, Chaponnier C, Brown RA. Myofibroblasts and mechano-regulation of connective tissue remodelling. Nat Rev Mol Cell Biol. 2002;3(5):349–63. Epub 2002/05/04. 10.1038/nrm809 nrm809 [pii]. . 11988769

[25] Meng N, Li Y, Zhang H, Sun XF. RECK, a novel matrix metalloproteinase regulator. Histol Histopathol. 2008;23(8):1003–10. Epub 2008/05/24. . 18498076 10.14670/HH-23.1003

[26] Oh J, Takahashi R, Kondo S, Mizoguchi A, Adachi E, Sasahara RM, et al The membrane-anchored MMP inhibitor RECK is a key regulator of extracellular matrix integrity and angiogenesis. Cell. 2001;107(6):789–800. Epub 2001/12/19. doi: S0092-8674(01)00597-9 [pii]. . 11747814 10.1016/s0092-8674(01)00597-9

[27] Chandana EP, Maeda Y, Ueda A, Kiyonari H, Oshima N, Yamamoto M, et al Involvement of the Reck tumor suppressor protein in maternal and embryonic vascular remodeling in mice. BMC developmental biology. 2010;10:84 Epub 2010/08/10. 10.1186/1471-213X-10-84 20691046 PMC2923112

[28] Miki T, Shamma A, Kitajima S, Takegami Y, Noda M, Nakashima Y, et al The ss1-integrin-dependent function of RECK in physiologic and tumor angiogenesis. Molecular cancer research: MCR. 2010;8(5):665–76. Epub 2010/04/22. 10.1158/1541-7786.MCR-09-0351 . 20407016

[29] Hatta M, Matsuzaki T, Morioka Y, Yoshida Y, Noda M. Density- and serum-dependent regulation of the Reck tumor suppressor in mouse embryo fibroblasts. Cell Signal. 2009;21(12):1885–93. Epub 2009/09/02. 10.1016/j.cellsig.2009.08.005 S0898-6568(09)00243-5 [pii]. . 19720143

[30] Massague J. TGFbeta signalling in context. Nat Rev Mol Cell Biol. 2012;13(10):616–30. Epub 2012/09/21. 10.1038/nrm3434 22992590 PMC4027049

[31] Grotendorst GR, Okochi H, Hayashi N. A novel transforming growth factor beta response element controls the expression of the connective tissue growth factor gene. Cell Growth Differ. 1996;7(4):469–80. Epub 1996/04/01. . 9052988

[32] Ignotz RA, Massague J. Transforming growth factor-beta stimulates the expression of fibronectin and collagen and their incorporation into the extracellular matrix. J Biol Chem. 1986;261(9):4337–45. Epub 1986/03/25. . 3456347

[33] Ignotz RA, Endo T, Massague J. Regulation of fibronectin and type I collagen mRNA levels by transforming growth factor-beta. J Biol Chem. 1987;262(14):6443–6. Epub 1987/05/15. . 3471760

[34] Massague J, Blain SW, Lo RS. TGFbeta signaling in growth control, cancer, and heritable disorders. Cell. 2000;103(2):295–309. Epub 2000/11/01. doi: S0092-8674(00)00121-5 [pii]. . 11057902 10.1016/s0092-8674(00)00121-5

[35] Droguett R, Cabello-Verrugio C, Santander C, Brandan E. TGF-beta receptors, in a Smad-independent manner, are required for terminal skeletal muscle differentiation. Exp Cell Res. 2010;316(15):2487–503. Epub 2010/05/18. 10.1016/j.yexcr.2010.04.031 S0014-4827(10)00202-8 [pii]. . 20471380

[36] Kocic J, Bugarski D, Santibanez JF. SMAD3 is essential for transforming growth factor-beta1-induced urokinase type plasminogen activator expression and migration in transformed keratinocytes. European journal of cancer. 2012;48(10):1550–7. Epub 2011/07/30. 10.1016/j.ejca.2011.06.043 . 21798735

[37] Jinnin M, Ihn H, Tamaki K. Characterization of SIS3, a novel specific inhibitor of Smad3, and its effect on transforming growth factor-beta1-induced extracellular matrix expression. Molecular pharmacology. 2006;69(2):597–607. Epub 2005/11/17. 10.1124/mol.105.017483 . 16288083

[38] Wrana JL, Attisano L, Carcamo J, Zentella A, Doody J, Laiho M, et al TGF beta signals through a heteromeric protein kinase receptor complex. Cell. 1992;71(6):1003–14. Epub 1992/12/11. doi: 0092-8674(92)90395-S [pii]. . 1333888 10.1016/0092-8674(92)90395-s

[39] Drobic V, Cunnington RH, Bedosky KM, Raizman JE, Elimban VV, Rattan SG, et al Differential and combined effects of cardiotrophin-1 and TGF-beta1 on cardiac myofibroblast proliferation and contraction. Am J Physiol Heart Circ Physiol. 2007;293(2):H1053–64. Epub 2007/05/08. doi: 00935.2006 [pii] 10.1152/ajpheart.00935.2006 . 17483238

[40] Liu L, Nishio N, Ito S, Tanaka Y, Isobe K. Negative regulation of GADD34 on myofibroblasts during cutaneous wound healing. Biomed Res Int. 2014;2014:137049 Epub 2014/09/12. 10.1155/2014/137049 25210702 PMC4156997

[41] Nishiyama T, Kii I, Kashima TG, Kikuchi Y, Ohazama A, Shimazaki M, et al Delayed re-epithelialization in periostin-deficient mice during cutaneous wound healing. PLoS One. 2011;6(4):e18410 Epub 2011/04/15. 10.1371/journal.pone.0018410 21490918 PMC3072397

[42] Romana-Souza B, Nascimento AP, Brum PC, Monte-Alto-Costa A. Deletion of the alpha2A/alpha2C-adrenoceptors accelerates cutaneous wound healing in mice. Int J Exp Pathol. 2014;95(5):330–41. Epub 2014/09/05. 10.1111/iep.12093 . 25186490 PMC4209925

[43] Gomes LR, Terra LF, Wailemann RA, Labriola L, Sogayar MC. TGF-beta1 modulates the homeostasis between MMPs and MMP inhibitors through p38 MAPK and ERK1/2 in highly invasive breast cancer cells. BMC cancer. 2012;12:26 Epub 2012/01/21. 10.1186/1471-2407-12-26 22260435 PMC3277461

[44] Kim NY, Lee JE, Chang HJ, Lim CS, Nam DH, Min BH, et al Gamma-irradiation enhances RECK protein levels in Panc-1 pancreatic cancer cells. Molecules and cells. 2008;25(1):105–11. Epub 2008/03/06. . 18319621

[45] Lee H, Lim C, Lee J, Kim N, Bang S, Lee H, et al TGF-beta signaling preserves RECK expression in activated pancreatic stellate cells. J Cell Biochem. 2008;104(3):1065–74. Epub 2008/02/27. 10.1002/jcb.21692 . 18300271

[46] Yuki K, Yoshida Y, Inagaki R, Hiai H, Noda M. E-cadherin-downregulation and RECK-upregulation are coupled in the non-malignant epithelial cell line MCF10A but not in multiple carcinoma-derived cell lines. Scientific reports. 2014;4:4568 Epub 2014/04/03. 10.1038/srep04568 24691523 PMC3972504

[47] Morioka Y, Monypenny J, Matsuzaki T, Shi S, Alexander DB, Kitayama H, et al The membrane-anchored metalloproteinase regulator RECK stabilizes focal adhesions and anterior-posterior polarity in fibroblasts. Oncogene. 2009;28(11):1454–64. Epub 2009/01/27. 10.1038/onc.2008.486 onc2008486 [pii]. . 19169281

[48] Park S, Lee C, Sabharwal P, Zhang M, Meyers CL, Sockanathan S. GDE2 promotes neurogenesis by glycosylphosphatidylinositol-anchor cleavage of RECK. Science. 2013;339(6117):324–8. Epub 2013/01/19. 10.1126/science.1231921 339/6117/324 [pii]. 23329048 PMC3644959

[49] Muraguchi T, Takegami Y, Ohtsuka T, Kitajima S, Chandana EP, Omura A, et al RECK modulates Notch signaling during cortical neurogenesis by regulating ADAM10 activity. Nat Neurosci. 2007;10(7):838–45. Epub 2007/06/15. doi: nn1922 [pii] 10.1038/nn1922 . 17558399

[50] Hong KJ, Wu DC, Cheng KH, Chen LT, Hung WC. RECK inhibits stemness gene expression and tumorigenicity of gastric cancer cells by suppressing ADAM-mediated Notch1 activation. J Cell Physiol. 2014;229(2):191–201. Epub 2013/07/25. 10.1002/jcp.24434 . 23881612

[51] Zanotti S, Canalis E. Notch signaling in skeletal health and disease. Eur J Endocrinol. 2013;168(6):R95–103. Epub 2013/04/05. 10.1530/EJE-13-0115 EJE-13-0115 [pii]. . 23554451 PMC4501254

[52] Gingras D, Bousquet-Gagnon N, Langlois S, Lachambre MP, Annabi B, Beliveau R. Activation of the extracellular signal-regulated protein kinase (ERK) cascade by membrane-type-1 matrix metalloproteinase (MT1-MMP). FEBS Lett. 2001;507(2):231–6. Epub 2001/10/31. doi: S0014-5793(01)02985-4 [pii]. . 11684104 10.1016/s0014-5793(01)02985-4

[53] Silveira Correa TC, Massaro RR, Brohem CA, Taboga SR, Lamers ML, Santos MF, et al RECK-mediated inhibition of glioma migration and invasion. J Cell Biochem. 2010;110(1):52–61. Epub 2010/02/04. 10.1002/jcb.22472 . 20127710

[54] Clark JC, Thomas DM, Choong PF, Dass CR. RECK—a newly discovered inhibitor of metastasis with prognostic significance in multiple forms of cancer. Cancer Metastasis Rev. 2007;26(3–4):675–83. Epub 2007/09/11. 10.1007/s10555-007-9093-8 . 17828469

[55] Takahashi C, Sheng Z, Horan TP, Kitayama H, Maki M, Hitomi K, et al Regulation of matrix metalloproteinase-9 and inhibition of tumor invasion by the membrane-anchored glycoprotein RECK. Proc Natl Acad Sci U S A. 1998;95(22):13221–6. Epub 1998/10/28. 9789069 10.1073/pnas.95.22.13221 PMC23764

[56] Mezzano V, Cabrera D, Vial C, Brandan E. Constitutively activated dystrophic muscle fibroblasts show a paradoxical response to TGF-beta and CTGF/CCN2. J Cell Commun Signal. 2007;1(3–4):205–17. Epub 2008/07/05. 10.1007/s12079-008-0018-2 18600480 PMC2443238

[57] Vangipuram M, Ting D, Kim S, Diaz R, Schule B. Skin punch biopsy explant culture for derivation of primary human fibroblasts. J Vis Exp. 2013;(77):e3779 Epub 2013/07/16. 10.3791/3779 . 23852182 PMC3731437

[58] Gutierrez J, Brandan E. A novel mechanism of sequestering fibroblast growth factor 2 by glypican in lipid rafts, allowing skeletal muscle differentiation. Mol Cell Biol. 2010;30(7):1634–49. Epub 2010/01/27. 10.1128/MCB.01164-09 MCB.01164-09 [pii]. 20100867 PMC2838066

