# Supplementary figures and images for: RECK-Mediated β1-Integrin Regulation by TGF-β1 Is Critical for Wound Contraction in Mice
Source: PLoS One. 2015 Aug 6;10(8):e0135005. doi: 10.1371/journal.pone.0135005 (PMC4527692; doi:10.1371/journal.pone.0135005)

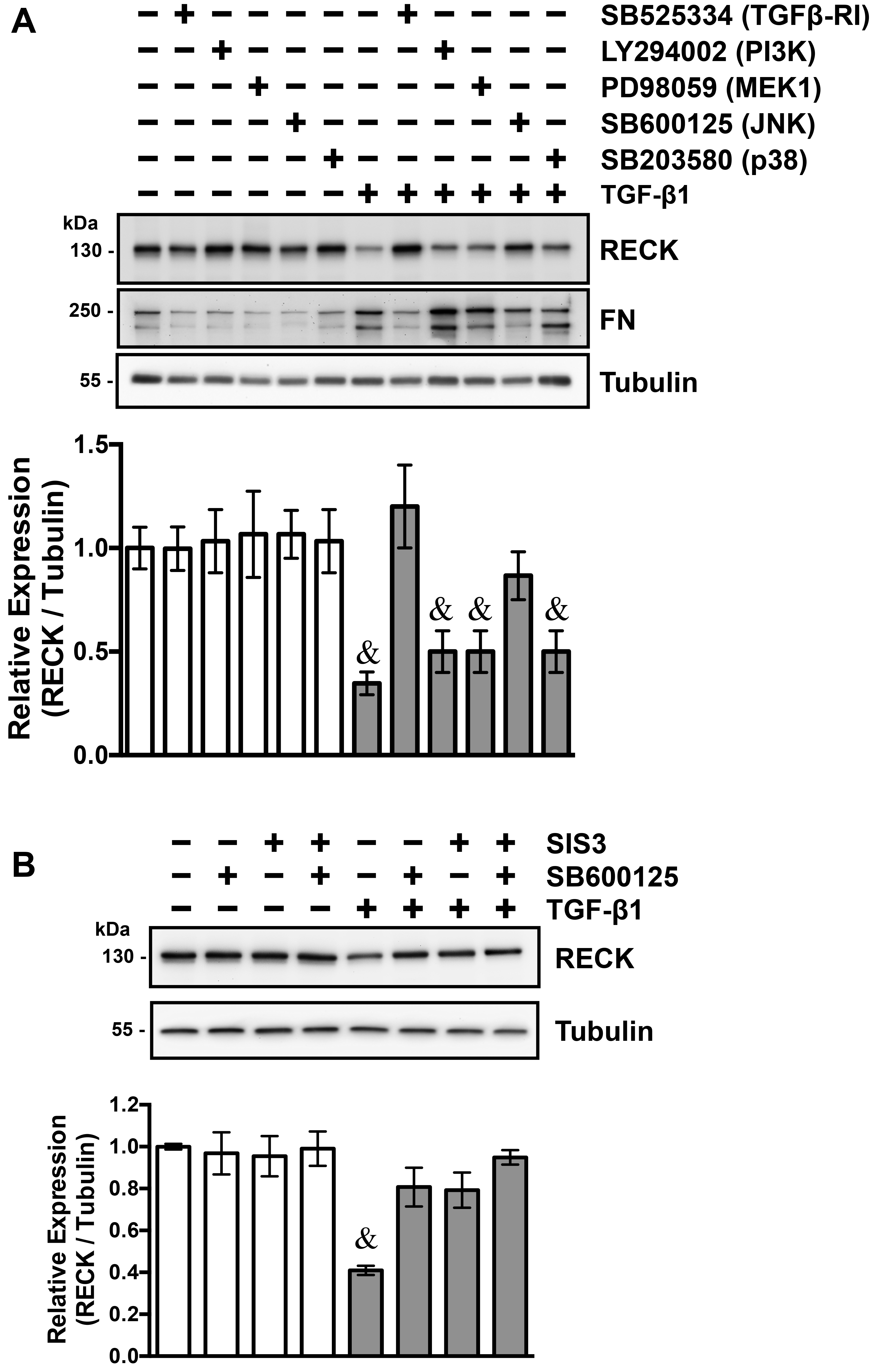

Supplement: S1 Fig — (A) NIH3T3 fibroblasts were pre-treated for 30 minutes with different inhibitors: TGF-β-RI kinase inhibitor SB525334, PI3K inhibitor LY294002, MEK1 inhibitor PD98059, JNK inhibitor SB600125 and p38 inhibitor SB203580. After the pre-treatment, fibroblasts were treated with 5 ng/ml of TGF-β1 for 24 hours or left untreated as a control. Western blot analysis of cell extracts were performed to determine the levels of RECK and FN. β-tubulin levels were used as a loading control. (B) NIH3T3 fibroblasts were pre-treated for 30 minutes with SIS3, a specific inhibitor of Smad-3 activation, and the JNK inhibitor SB600125; fibroblasts were treated with either inhibitor alone or in combination. Cells were then treated with 5 ng/ml of TGF-β1 for 24 hours, or left untreated as a control. Western blot analysis of cell extracts were performed to determine the levels of RECK and FN. Tubulin levels were used as a loading control. The quantifications shown in A and B are derived from two independent experiments. Statistical significance was assessed using two-way ANOVA and a Bonferroni multiple-comparison post hoc test. &, P<0.05 relative to TGF-β1 untreated fibroblasts. (TIF) [file pone.0135005.s001.tif]

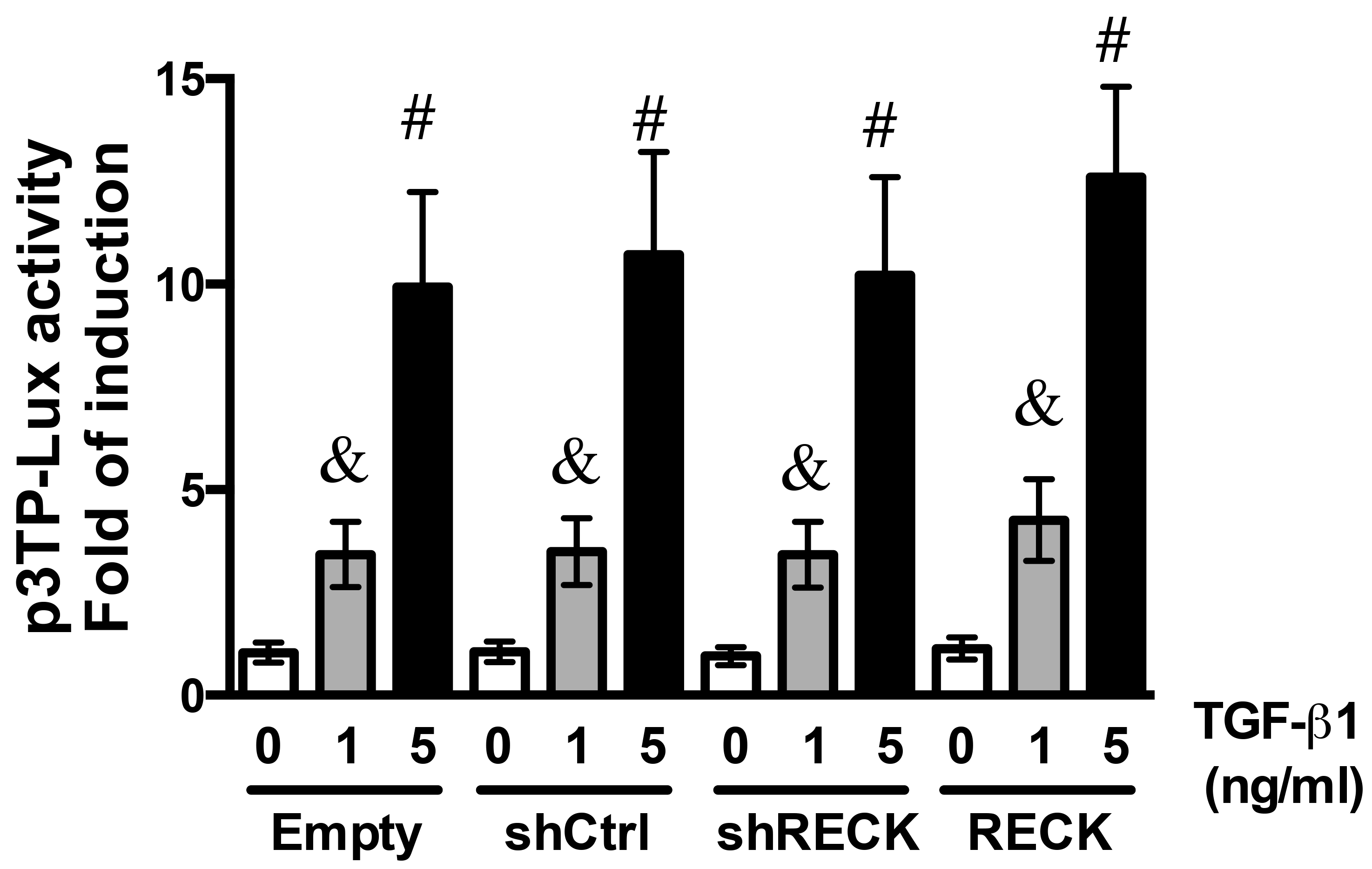

Supplement: S2 Fig — NIH3T3 fibroblasts were transiently co-transfected with the TGF-β1 reporter system p3TPLux/pRL and: with shRECK, with a RECK overexpression vector or with an empty vector as control. 24 h post-transfection, the cells were incubated with TGF-β1 at the indicated concentrations. Luciferase activity was determined after 24 hours of TGF-β1 treatment. The quantifications is from two independent experiments. Statistical significance was assessed using two-way ANOVA and a Bonferroni multiple-comparison post hoc test. &, P<0.05 relative to 0 ng/ml TGF-β1; #, P<0.05 P<0.05 relative to 1 ng/ml TGF-β1 in each case. (TIF) [file pone.0135005.s002.tif]

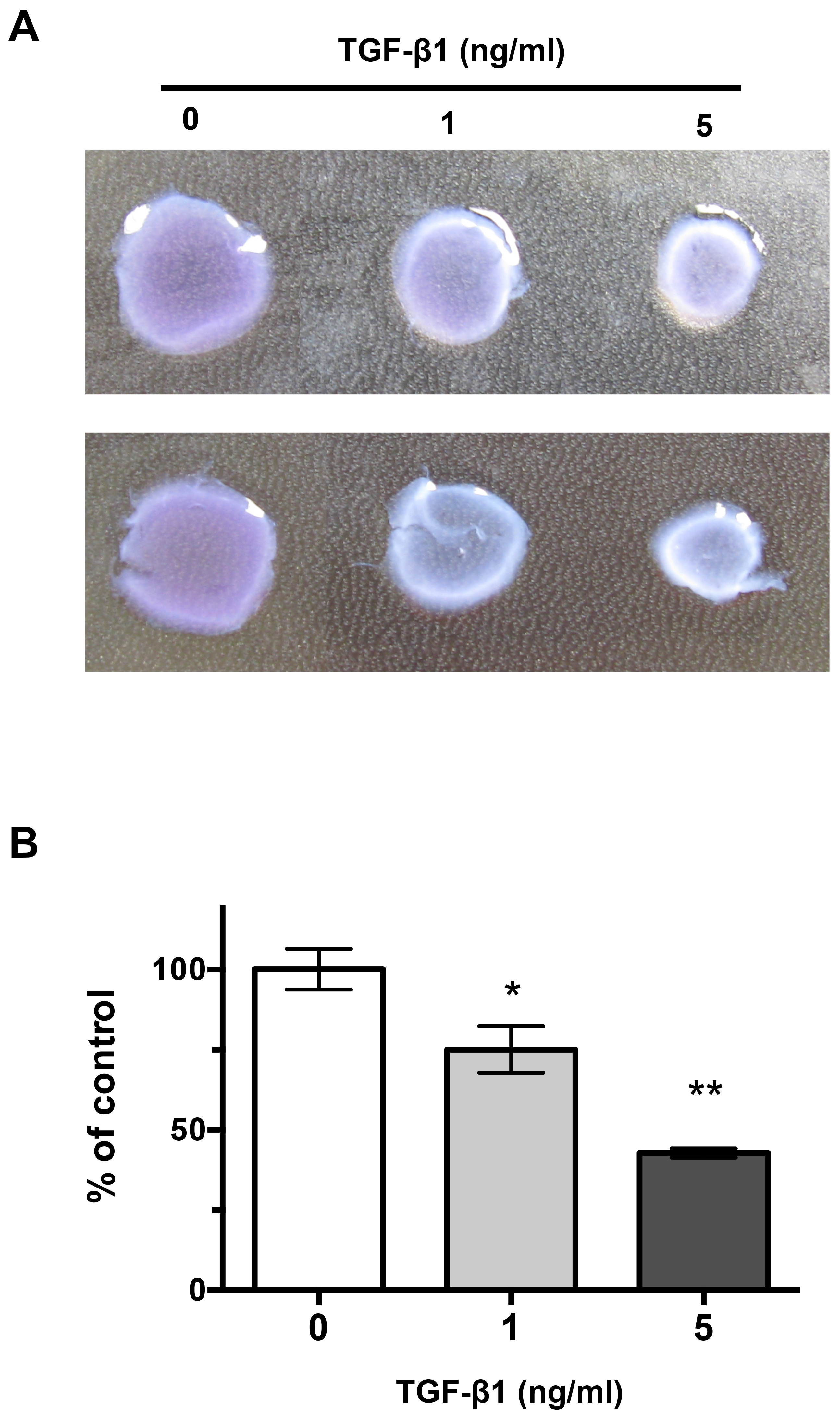

Supplement: S3 Fig — (A) NIH3T3 fibroblasts were subjected to a collagen contraction assay, as described in Fig 6A. After 24 hours of treatment with TGF-β1 at two different concentrations, as indicated, the 3D floating matrices were photographed. Representative images are shown. (B) The volume of the contracted matrices obtained was measured immediately after being released at the end of the assay and graphed as a percentage of the TGF-β1 untreated fibroblast volume. (TIF) [file pone.0135005.s003.tif]
